# Artemisinin Resistance and Stage Dependency of Parasite Clearance in Falciparum Malaria

**DOI:** 10.1093/infdis/jiy673

**Published:** 2019-01-17

**Authors:** Benjamas Intharabut, Hugh W Kingston, Ketsanee Srinamon, Elizabeth A Ashley, Mallika Imwong, Mehul Dhorda, Charles Woodrow, Kasia Stepniewska, Kamolrat Silamut, Nicholas P J Day, Arjen M Dondorp, Nicholas J White, Tracking Resistance to Artemisinin Collaboration

**Affiliations:** 1 Mahidol-Oxford Tropical Medicine Research Unit, Faculty of Tropical Medicine, Mahidol University, Bangkok, Thailand; 2 Department of Clinical Tropical Medicine, Faculty of Tropical Medicine, Mahidol University, Bangkok, Thailand; 3 Department of Molecular Tropical Medicine and Genetics, Faculty of Tropical Medicine, Mahidol University, Bangkok, Thailand; 4 WorldWide Antimalarial Resistance Network, Asia Regional Centre, Bangkok, Thailand; 5 Centre for Tropical Medicine and Global Health, Nuffield Department of Clinical Medicine, Churchill Hospital, Oxford, United Kingdom; 6 Myanmar Oxford Clinical Research Unit, Yangon, Myanmar

**Keywords:** Falciparum malaria, microscopy, artemisinin resistance, parasite clearance, parasite stage

## Abstract

**Background:**

Artemisinin resistance in falciparum malaria is associated with *kelch13* propeller mutations, reduced ring stage parasite killing, and, consequently, slow parasite clearance. We assessed how parasite age affects parasite clearance in artemisinin resistance.

**Methods:**

Developmental stages of *Plasmodium falciparum* parasites on blood films performed at hospital admission and their *kelch13* genotypes were assessed for 816 patients enrolled in a multinational clinical trial of artemisinin combination therapy.

**Results:**

Early changes in parasitemia level (ie, 0–6 hours after admission) were determined mainly by modal stage of asexual parasite development, whereas the subsequent log-linear decline was determined mainly by *kelch13* propeller mutations. Older circulating parasites on admission were associated with more-rapid parasite clearance, particularly in *kelch13* mutant infections. The geometric mean parasite clearance half-life decreased by 11.6% (95% CI 3.4%–19.1%) in *kelch13* wild-type infections and by 30% (95% CI 17.8%–40.4%) in *kelch13* mutant infections as the mean age of circulating parasites rose from 3 to 21 hours.

**Conclusion:**

Following the start of antimalarial treatment, ongoing parasite sequestration and schizogony both affect initial changes in parasitemia. The greater dependency of parasite clearance half-life on parasite age in artemisinin resistant infections is consistent with ring stage resistance and consequent parasite clearance by sequestration. The stage of parasite development should be incorporated in individual assessments of artemisinin resistance.

## Teaser

The effect of parasite stage of development on the parasite clearance curve was investigated in areas affected by artemisinin resistance. Parasite stage shapes the early part of the clearance curve and also influences the subsequent log-linear decline.

Parasite clearance in acute malaria is a complex process determined by host, parasite, and drug treatment factors [1, 2]. Indices derived from the parasite clearance curve, calculated as the plot of the parasite count against the time after starting antimalarial drug treatment, are used to assess therapeutic responses in falciparum malaria. Standardized methods have been developed to analyze parasite clearance curves following treatment with artemisinin or its derivatives and to calculate the half-life of the log-linear decline (PC_1/2_) [1]. This measure is particularly useful in assessing artemisinin-containing regimens, as these drugs have a much greater effect on circulating ring stage parasites than do other antimalarials in current use [2].

The PC_1/2_ has been used widely as an outcome measure in clinical trials and epidemiological assessments of artemisinin efficacy [1, 3]. A decrease in the first order parasite clearance rate (ie, an increase in PC_1/2_) is the pharmacodynamic hallmark of artemisinin resistance, which is associated with mutations in the “propeller region” of the *kelch13* gene on chromosome 13 [4]. Deduction [3] supported by modeling [5] and in vitro studies indicated that artemisinin resistance in *P. falciparum* reflected reduced asexual parasite ring stage susceptibility [4]. In addition to antimalarial drug resistance and inadequate drug exposure, other factors may affect the parasite clearance curve. For example, patients with asplenia have very slow parasite clearance following treatment with artemisinin derivatives as it is the spleen that removes either the entire infected erythrocyte or extracts the dead parasites from infected erythrocytes (a process called pitting) [2, 6]. Whereas patients with acute malaria may present with a wide range of parasitemia levels (up to a 5-log_10_ difference), there is little relation between the baseline parasite count and the parasite clearance rate or PC_1/2_ [2, 7]. Immunity is an important contributor to the overall therapeutic response, but when assessed serologically or by epidemiological background, it is associated with only modest reductions in PC_1/2_ of 0.16–0.65 hours [8].

Changes in parasitemia level may also result from parasite factors that are unrelated to drug susceptibility, notably the stage and synchronicity of the infection before antimalarial treatment starts [9]. In some patients, there is a lag phase after starting treatment, in which the parasite count is static or increases before beginning the log-linear decline [1]. Occasionally, a marked increase of up to 10-fold or more in the parasitemia level occurs after starting treatment, as synchronous deep vascular schizogony liberates merozoites and a wave of new circulating ring form–infected erythrocytes appear in the circulation [10]. Such increases in the parasitemia level are less common following treatment with artemisinin derivatives, compared with previous observations for the other, less potent antimalarial drugs, which have relatively little effect on ring stage parasites [11]. The opposite phenomenon may also occur sometimes, in which there is an initial, more rapid decrease, presumably resulting from synchronous sequestration, followed by a less steep log-linear descent.

It was hypothesized that the stage of parasite development is an important covariate in the analysis of parasite clearance curve data, and because of the stage specificity of artemisinin resistance, its effect may differ in resistant strains. The effects of artemisinin resistance on these early post-treatment changes in parasitemia level and their relationship to the stage of parasite development were evaluated in a large multicenter trial [12] conducted in areas affected by artemisinin resistance.

## METHODS

### Patients

The Tracking Resistance to Artemisinin Collaboration (TRAC) study was a multicenter, open-label study, which started in 2011, of adults and children with acute uncomplicated falciparum malaria. TRAC aimed to map the extent and severity of artemisinin resistance [12]. Full details of enrollment, treatment, and outcomes have been published already [12]. Patients with uncomplicated malaria were enrolled in Cambodia, Thailand, Laos, Vietnam, Myanmar, Bangladesh, India, Nigeria, Kenya, and the Democratic Republic of the Congo (DRC). Patients received either 2 or 4 mg/kg oral artesunate (AS) for 3 days, followed by a full 3-day course of artemisinin combination therapy, except in the DRC, where they received either artemether–lumefantrine (AL) for 3 days only or 4 mg/kg AS for 3 days followed by 3 days of AL, and in Kenya, where they received 2 mg/kg AS for 7 days. The protocol was approved by the Oxford Tropical Research Ethics Committee and the institutional review board, national ethics committee, or both for each study site.

### Laboratory Procedures

Parasite counts and hematocrits were assessed at the time the antimalarial was first taken (baseline); 4, 6, 8, and 12 hours after baseline and every 6 hours thereafter until consecutive parasite-negative blood films were reported; and 72 hours after baseline in all cases. Malaria parasite counts on Giemsa-stained thin films were recorded as the number of parasites per 1000 red blood cells (RBCs), and counts on thick films were recorded as the number of parasites per 500 white blood cells (WBCs). The parasitemia level (parasite density), recorded as the number of parasites/µL, was calculated for thin film counts by using the following formula: [parasite count per 1000 RBCs] * 125.6 * [hematocrit] this assumes a mean cell volume of 80 fL. For thick film counts, the following formula was used: [parasite count per 500 WBCs] * 16 (this assumes a WBC count of 8000 WBCs/µL) [13]. Parasite counts were performed at the study sites, and counts at baseline and at 72 hours were rechecked centrally. Full details of the quality control methods have been published previously [12]. The parasite stage of asexual development was assessed at the Mahidol-Oxford Tropical Medicine Research Unit (Bangkok, Thailand) on the baseline blood film, based on the method of Silamut et al [14], where parasites are described as tiny rings (TRs), small rings (SRs), or large rings (LRs); early trophozoites (ETs), middle trophozoites (MTs), or late trophozoites (LTs); schizonts (Shs); and gametocytes [14]. If the parasite density was sufficient, 100 parasites were staged on the thin film. Microscopists were unaware of the genotyping or PC_1/2_ data.

Genotyping for *kelch13* mutations was performed as described previously [12]. Nonsynonymous mutations at positions ≥441 in the gene, except those at position 578 (mutations here are not associated with in vitro resistance or a prolonged PC_1/2_), were considered artemisinin resistant; parasites with these mutations are hereafter referred to as *kelch13* mutants [15]. Patients with mixed-genotype infections were excluded.

### Statistical Methods

Statistical analyses were performed using Stata, version 14.0 (StataCorp, College Station, TX). The modal developmental stage of parasites present on admission was defined as the most frequently occurring stage seen for each patient, with the older stage used in the case of a tie. Parasite clearance half-lives were calculated using the WorldWide Antimalarial Resistance Network parasite clearance estimator [1]. The percentage reduction in parasitemia at *t* hours was calculated as 100 * [(baseline parasitemia level – parasitemia level at *t* hours) / (baseline parasitemia level)]. The relative parasitemia level was calculated as [parasitemia level at *t* hours]/[baseline parasitemia level]. Differences in relative parasitemia at time points grouped by modal stage or genotype were compared using linear regression that adjusted for site and treatment. Because the age of circulating parasites is a circular quantity (ie, the schizont stage is adjacent to the tiny ring stage), the circular mean age of the parasites staged on each patient’s baseline blood film was calculated for each patient, assuming the following ages: TR stage, 3 hours; SR stage, 11 hours; LR stage, 21 hours; ET stage, 28 hours; MT stage, 32 hours; LT stage, 36 hours; and Sh stage, 43 hours [16].

Associations between baseline characteristics (ie, parasitemia level, *kelch13* genotype, and modal stage) were explored using regression models or partial correlation, adjusted for study site. Trends between the PC_1/2_ or the reduction in parasitemia level at 6 hours and modal stage were assessed using parametric (Pearson) partial correlation, adjusted for site and initial drug treatment. The 6-hour time point was chosen because it was reported previously to be the median duration of the lag phase [7]. Logistic regression was used to assess whether *kelch13* genotype was associated with an increase in parasitemia level at 6 hours, adjusted for site and initial drug treatment. Linear regression was used to assess the associations between the log_10_ (PC_1/2_) or the ratio of the parasitemia level at 6 hours to the level at baseline and the circular mean age of the circulating parasites, *kelch13* genotype, and drug treatment, with adjustment for site.

Individual parasite clearance profiles could be divided into 3 basic types: (1) a lead phase, in which a more rapid initial reduction in parasite density preceded a less rapid log-linear descent; (2) a lag phase, in which the parasite count rose or had an initial, less steep decrease before decreasing; and (3) a phase in which parasite counts decreased log linearly throughout. As a simple method of quantifying this, 2 linear regression models for individual patients’ log parasitemia levels between 0 and 24 hours were fitted. The first model included only the time variable *t* as a covariate, and the second included *t* and a second variable, τ, to account for a change in gradient after 6 hours. The variable τ was defined as follows: τ = *t* – 6 if t > 6 and τ = 0 if *t* ≤ 6. A likelihood ratio test was used to compare the 2 models to see whether addition of τ significantly improved the fit (*P* < .05). If it did and the coefficient for τ was >0, then the profile was called a “lead,” whereas if τ significantly improved the fit but the coefficient for τ was <0, the profile was called a “lag.” If τ did not improve the fit significantly, then the profile had neither lag nor lead phases. Proportions of parasites in TR, SR, or LR stages were logit transformed for partial correlation with the shape of profile (ordinal; lag, no lag, lead), with adjustment for site and treatment; when the proportion was 0, this was replaced with 0.005 (ie, one half of the minimum detectable proportion).

## RESULTS

Of 1241 patients with uncomplicated falciparum malaria enrolled into the TRAC study, parasite developmental staging data were available for 991 (80%). Of these, both parasite clearance data and *kelch13* mutation status were available for 902 (91%). Because of heterozygosity in the *kelch13* genotype (ie, mixed infection), 86 patients were excluded, leaving data from 816 of 1241 patients (66%) for analysis. Of these patients, 8.2% (67 of 816) were from Africa, and the remainder were from Asia. Nearly all patients (789 of 816 [97%]) had ring stages as the modal stage of their presenting peripheral parasitemia (Table 1**).** The median percentage of tiny rings was 21% (range, 0%–99%). The median circular mean age of the circulating parasites was 9.9 hours (range, 3.1–30 hours). Older as opposed to younger modal stages were associated with lower median parasitemia levels in *kelch13* wild-type infections (Table 1). There was no significant association between modal stage and *kelch13* mutation status after adjustment for site (ordinal logistic regression model, *P* = .403). In a linear regression model for baseline log_10_ parasitemia level adjusted for site, the geometric mean baseline parasitemia level was 16.8% lower (95% confidence interval [CI], 2.3%–29.2%) in *kelch13* mutant infections as compared to wild-type infections.

**Table 1.** Parasite Clearance Measures and Stage of *Plasmodium falciparum* Development on the **Admission** Blood Smear

Modal Stage of Development^a^	Patients, No.		Baseline Parasite Count, Parasites/µL^b^		Percentage Reduction in Parasitemia Level at 6 h		PC_1/2_, h^b^	
	*kelch13* WT	*kelch13* Mutant	*kelch13* WT	*kelch13* Mutant	*kelch13* WT	*kelch13* Mutant	*kelch13* WT	*kelch13* Mutant
Tiny rings	189	87	66 317 (36 173–105 504)	55 892 (33 410–101 422)	27 (5–42)	22 (−2–39)	2.5 (2–3.2)	6.8 (5.5–8.3)
Small rings	232	127	59 974 (30 144–100 794)	50 240 (30 521–87 543)	33 (11–55)	27 (9–44)	2.7 (2.1–3.4)	6.2 (5–7.6)
Large rings	94	59	50 240 (30 144–101 108)	45 216 (23 236–70 838)	65 (40–83)	56 (32–75)	2.4 (1.7–3.1)	5.5 (4.5–6.5)
Early trophozoites	23	3	30 772 (20 096–52 752)	29 390 (27 130–32 154)	73 (55–92)	82 (67–92)	1.9 (1.6–2.5)	3.9 (2.6–4.3)
Middle trophozoites	1	0	12 058	NA	99	NA	0.8	NA
*P*_trend_^c^			.001	.20	<.001	<.001	.009	<.001

Data are median values (interquartile ranges), unless otherwise indicated.

Abbreviations: NA, not applicable; PC_1/2,_ parasite clearance half-life; WT wild type.

^a^No patient had a modal stage of late trophozoites or schizonts.

^b^Variables were log transformed for assesment of *P*_trend_.

^c^Derived from partial correlation of the variable of interest with of stage of development (ordinal), adjusted for site as a factor and also adjusted for drug in analyzing parasitemia reduction at 6 hours and PC_1/2_.

### Early Changes in Parasite Density

By 6 hours after starting treatment, increases in parasitemia levels occurred in 18% of patients (50 of 274) with *kelch13* mutant infections and 12% (60 of 489) with wild-type infections; this difference was not significant after adjustment for site and treatment (logistic regression model *P* = .79). Stage and not *kelch13* mutation status was the main factor affecting the very early changes in parasitemia level. In both wild-type and *kelch13* mutant infections, the median percentage reduction in parasitemia level at 6 hours increased with increasing baseline modal parasite age (Table 1). In a linear regression model (model 1) that adjusted for site, an older circular mean parasite age predicted a larger reduction in parasitemia level at 6 hours (Table 2), whereas *kelch13* genotype was not a significant predictor. Addition of an interaction term between *kelch13* genotype and stage was not significant (*P* > .05), and addition of initial drug treatment or patient age did not improve the fit of the model (*P* > .05).

**Table 2.** Linear Regression Modeling of Parasite Clearance and Stage of Parasite Development

Model	Coefficient Estimate (95% CI)	Percentage Change in Geometric Mean PC_1/2_ (95% CI)
Model 1. Prediction of 6 h to 0 h parasitemia ratio (n = 763; R^2^ = 0.18)		
Genotype *kelch13* mutant	−0.04 (−0.13 to –.05)	…
Circular mean age (in h)	−0.031 (−0.036 to −0.026)	…
Model 2. Prediction of PC_1/2_ (n = 816; R^2^ = 0.60)		
*kelch13* mutant	0.4 (.34 to .46)	150 (118 to 186)
Circular mean age (in h) (*kelch13* wild type)	−0.003 (−0.005 to −0.001)	−0.68 (−1.17 to −0.19)
Circular mean age (in h) (*kelch13* mutant)	−0.009 (−0.012 to −0.005)	−1.96 (−2.83 to −1.08)
Log_10_ parasitemia level (in parasites/µL)	0.05 (0.01 to .08)	10.9 (3 to 19.5)
AL	0.11 (.02 to .2)	29 (4 to 60.1)
AS4	−0.02 (−.05 to .01)	−4.57 (−10 to 1.19)

Models were adjusted for site. The reference group for model 1 is *kelch13* wild type and that for model 2 is *kelch13* wild type treated with AS2.

Abbreviations: AL, artemether–lumefantrine; AS2, 2 mg/kg artesunate; AS4, 4 mg/kg artesunate; PC_1/2_ parasite clearance half-life.

The parasite clearance curves were classified as lag in 50% of *kelch13* wild-type and 23% of mutant infections; as neither lag nor lead in 45% and 64%, respectively; and as lead in 5% and 12%, respectively (Table 3). The highest proportion of tiny rings on admission blood films was in patients with a lag phase, while the smallest was in patients with a lead phase (Table 3). The reverse trend was observed for the proportion of large rings.

**Table 3.** Association Between Stage of Parasite Development at Admission and Subsequent Shape of the Parasite Clearance Profile

Variable	Infections, No. (%)^a^	TR	SR	LR
*kelch13* wild type (n = 418)				
Lag	209 (50)	32 (9–67)	51 (23–72)	2 (0–12)
No lag	188 (45)	15 (2–55)	38 (17–65)	7 (1–35)
Lead	21 (5)	0 (0–7)	42 (5–62)	30 (12–52)
*P*_trend_^b^		<.001	<.001	<.001
*kelch13* mutant (n = 273)				
Lag	64 (23)	34 (6 to 72)	39 (22–69)	5 (1–24)
No lag	175 (64)	15 (3 to 54)	46 (24–68)	9 (2–38)
Lead	34 (12)	3 (0 to 42)	33 (10–48)	38 (5–61)
*P*_trend_^b^		<.001	.21	<.001

Data are median percentages (interquartile range) of each stage, unless otherwise indicated.

Abbreviations: LR, large rings; SR, small rings; TR, tiny rings.

^a^Because of rounding, percentages might not sum to 100.

^b^For partial correlation between the shape of the profile (ordinal; lag, no lag, lead) and the logit-transformed proportion of stage, adjusted for initial treatment and site.

### Effect of Parasite Stage of Development on Early Parasite Clearance

**Figure 1.**
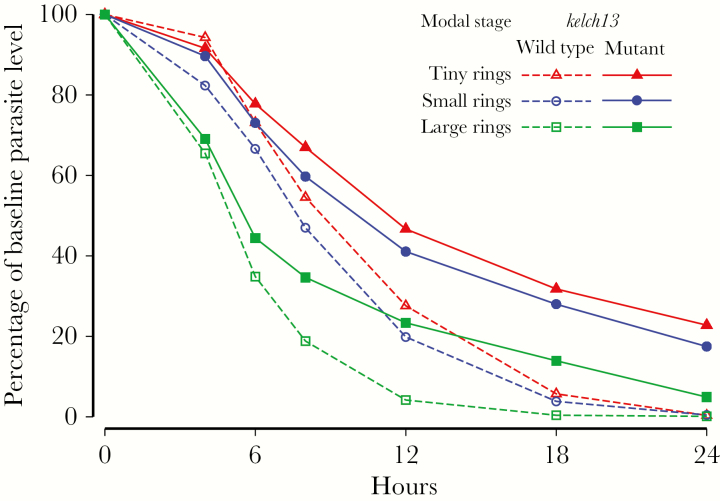
Comparison of the parasite counts over time as a proportion of the baseline parasite count, by modal stage of parasite development, in patients with artemisinin-susceptible and artemisinin-resistant *Plasmodium falciparum* malaria. Parasite counts after the first parasite-negative slide were replaced with a value of 0.

The profile of parasitemia level versus time differed depending on the parasite stage at admission. Early declines in parasite density were much greater in patients presenting with a predominance of more-mature parasites (Figure 1). In both *kelch13* wild-type and *kelch13* mutant infections, significant differences in relative parasitemia levels were observed at all time points between 4 and 24 hours when comparing patients with an initial modal stage of TRs or SRs to those with an initial modal stage of LRs, after adjustment for site and initial drug treatment (*P* < .05). Comparison of TR and SR stages revealed differences at 6, 12, and 18 hours in *kelch13* wild-type infections and differences at 6 and 12 hours in *kelch13* mutant infections, after adjustment for site and initial drug treatment (Figure 1).

Relative parasitemia levels decreased less rapidly in *kelch13* mutant infections than in wild-type infections; there were significant differences from 18 hours onward if TRs, SRs, or LRs composed the initial modal stage, after adjustment for site and initial drug treatment (Figure 1).

### PC_1/2_

The geometric mean PC_1/2_ in patients with *kelch13* wild-type infections was 2.5 hours (95% CI, 2.4–2.6), compared with 5.9 hours (95% CI, 5.7–6.2) in those with *kelch13* mutant infections. This difference remained significant after adjustment for site and treatment (*P* < .001). There was little change in the PC_1/2_ as the modal parasite age increased from TRs to LRs in the *kelch13* wild-type (ie, artemisinin-susceptible) infections, although in the small group of patients (n = 23) presenting with a modal stage of early trophozoites, the PC_1/2_ was markedly shorter (Table 1). In contrast, in patients with *kelch13* mutant infections, there was progressive shortening of the PC_1/2_ with increasing parasite age at presentation. In patients with *kelch13* mutant infections, the proportion with a PC_1/2_ below the threshold of 5 hours, which is widely used to define the artemisinin resistance phenotype [17], was 17% (15 of 88) among those in whom TRs predominated, 25% (32 of 127) among those in whom SRs predominated, and 34% (20 of 59) among those in whom LRs predominated.

In a linear regression model (Table 2), *kelch13* wild-type genotype, older circular mean age, and lower log_10_ parasitemia level were all significant predictors of a shorter log_10_ PC_1/2_, after adjustment for site and drug treatment. Addition of an interaction term between circular mean age of the staged parasites and *kelch13* genotype was significant: the decrease in the log_10_ PC_1/2_ in *kelch13* mutant infections became greater as the circular mean age increased (Table 2). Addition of an interaction term between *kelch13* genotype and parasitemia level or between circular mean age and parasitemia level did not improve model fit significantly. As the mean age of circulating parasites at admission increased from 3 to 21 hours (the mean ages of TR and LR stages, respectively), linear regression estimated that the geometric mean PC_1/2_ decreased by 11.6% (95% CI 3.4%–19.1%) in *kelch13* wild-type infections and by 30% (95%CI 17.8%–40.4%) in *kelch13* mutant infections.

## DISCUSSION

The parasite clearance profile in falciparum malaria reflects the net result of several different processes, which change with time. Before antimalarial treatment, new, young ring stage parasites enter the circulation while deep vascular schizogony continues, and older ring stages disappear from the circulation as they mature and the infected red cells sequester. After starting treatment, these processes continue for several hours until the parasites are affected by the antimalarial drug treatment. The relative contributions of these different processes to the changing parasite densities depend on the age distribution of the infecting parasites at presentation to medical attention [2]. In vitro drug-susceptibility assays show that, for most of the antimalarial drugs, the greatest antiparasitic activity is on the mature trophozoites (which, in *P. falciparum* malaria, are sequestered in venules and capillaries and therefore unseen by the microscopist) [18]. The artemisinin derivatives differ in having significant ring stage activity, as well. This results in more-rapid parasite clearance as the circulating parasites are damaged and removed, mainly by the spleen, and it is this property that largely explains the life-saving benefit of these drugs [19–21].

This study quantified in acute *P. falciparum* malaria the relative contributions of parasite stage of development to the early changes in parasite density that follow the start of treatment and to the subsequent log-linear decline. It assessed how this is affected by artemisinin resistance, and it showed how these interactions affect individual assessments of artemisinin resistance in vivo. The main findings are that the stage of asexual parasite development determines the early changes in parasitemia level following the start of treatment and, particularly in the case of artemisinin-resistant infections, has a significant effect on the subsequent rate of log-linear decline in parasitemia level. The stage of development therefore affects the measures of parasite clearance used to assess antimalarial drugs in general and artemisinin resistance in particular.

In the first 6 hours after starting treatment, reductions in the parasitemia level were slower, and a lag phase was more common when very young parasites predominated in both susceptible and resistant infections. These findings likely reflect input of new parasites from ongoing schizogony. Conversely, when older parasites predominated, the reduction in parasitemia level at 6 hours was greater, and a larger proportion of infections had an initial steeper decrease in parasitemia level (lead phase), findings consistent with early sequestration. Thus, asexual cycle stage of parasite development is one of the determinants of the initial treatment “response” [11]. Its effect on early changes in parasitemia level is much greater than that of artemisinin resistance. As the modal age increased from TRs to ETs, the median reduction in parasitemia level at 6 hours increased approximately 3-fold, from 27% to 73% whereas *kelch13* mutation status had no significant effect at this time point (model 1; Table 2). The lack of an effect of *kelch13* genotype on early reductions in the parasitemia level reflects the times taken for the artemisinin derivatives to reach parasiticidal levels, for these drugs to damage or kill the circulating malaria parasites, and for the spleen to remove them. Laboratory studies indicate little parasite pitting before 2 hours following drug exposure [22]. Thus, it is schizogony resulting in new RBCs becoming infected and sequestration causing clearance of older stages, rather than parasite killing, that are the dominant processes determining parasite densities in the first hours following the start of antimalarial treatment.

The slope of the log-linear decrease in parasitemia level, typically expressed as the PC_1/2_, is used widely for assessing treatment responses to artemisinin antimalarials. The *kelch13* mutant genotype is associated with a markedly prolonged PC_1/2_ [3, 4, 12]. In our data set, in *kelch13* mutant-type (ie, artemisinin-resistant) infections, PC_1/2_ was estimated to be 2.5 times longer than in *kelch13* wild-type (ie, artemisinin-susceptible) infections. In *kelch13* wild type infections (artemisinin sensitive) the PC_½_ was relatively independent of parasite stage. In marked contrast in *kelch13* mutant infections (artemisinin resistant) the slope was dependent on the stage of parasite development, with regression modeling indicating a decrease in PC_½_ as modal age increased. These findings are most likely explained by variation in the susceptibility of different stages of parasite development to artemisinins, with artemisinin resistance disproportionately affecting younger stages [4, 5, 23, 24], whereas sequestration causes initial clearance of more-mature parasites irrespective of resistance. In artemisinin-resistant infections, sequestration contributes significantly to parasite clearance during the first day of treatment, as it does following treatment with other antimalarial drugs, such as quinine, because a large proportion of circulating parasites continue to mature and then sequester, whereas in artemisinin-susceptible infections, relatively few circulating parasites survive the drug effect to do this [25]. Although artemisinin resistance is the main determinant of an increased PC_1/2,_ inclusion of parasite stage of development in the analysis permits evaluation of stage-specific drug effects and will reduce misclassification of the artemisinin resistance phenotype in vivo. In this study, patients presenting with *kelch13* mutant infections were approximately twice as likely to have a PC_1/2_ below the threshold of 5 hours when LR as compared with TR was the modal stage at presentation. Thus, in individual pharmacodynamic or molecular genotyping assessments, the stage of parasite development is an important confounder. Assessment of stage of development on blood films is simple to perform and robust [14]. Specifically, in investigating an individual case of suspected resistance to artemisinin (or indeed to any antimalarial with significant ring stage effects), parasite clearance rates may still be rapid if more-mature parasites predominate on the pretreatment blood film. Along with the results from serological assessments conducted in the same patient population [8], these findings do not support recent suggestions that immunity is the main determinant of parasite clearance rates [26].

Limitations of morphological assessment of parasite stage include observer misidentification of the correct stage and variation in the appearance of parasites of different strains [14, 27]. Only data from patients with uncomplicated malaria were assessed in this study, but there is no reason to suppose that a different relationship between stage and the lag phase would exist in severe malaria.

The shape and properties of the parasite clearance curve depend on the stage distribution of parasites before treatment. Initial changes in the parasitemia level following treatment are principally due to the stage-dependent processes of schizogony and sequestration, with little impact from artemisinin treatment. In individual assessments of artemisinin resistance, parasite stage should be assessed to avoid misclassifying infections in which older parasites predominate as being susceptible.

## Notes

***Acknowledgments.*** We thank the patients for agreeing to participate in these studies, our colleagues who assisted in patient recruitment, and the investigators of the TRAC study, including Prof Abul Faiz, Dr Pharath Lim, Dr Rick Fairhurst, Dr Chea Nguon, Dr Steffen Borrmann, Prof Mayfong Mayxay, Dr Ye Htut, Dr Olugbenga A. Mokuolu, Dr Marie Onyamboko, Dr Caterina Fanello, Prof François Nosten, Prof Sasithon Pukrittayakamee, Dr Rupam Tripura, and Prof Tran Tinh Hien.

***Disclaimer.*** The views expressed and information contained in this article are not necessarily those of or endorsed by the UK Department for International Development, which can accept no responsibility for such views or for any reliance place on them.

***Financial support.*** This work was supported by the UK Department for International Development and the Wellcome Trust.

***Potential conflicts of interest.*** All authors: No reported conflicts of interest. All authors have submitted the ICMJE Form for Disclosure of Potential Conflicts of Interest. Conflicts that the editors consider relevant to the content of the manuscript have been disclosed.
